# Exudative pericarditis in the evolution of a diffuse large B-cell lymphoma

**DOI:** 10.5830/CVJA-2015-024

**Published:** 2015

**Authors:** Cristina Bagacean, Dana Pop, Mihnea Zdrenghea, Adrian Tempescul, Jean-Christophe Ianotto, Veronique Marion, Dana Pop, Mihnea Zdrenghea

**Affiliations:** Iuliu Hatieganu University of Medicine and Pharmacy, Cluj-Napoca, Romania; Iuliu Hatieganu University of Medicine and Pharmacy, Cluj-Napoca, Romania; Iuliu Hatieganu University of Medicine and Pharmacy, Cluj-Napoca, Romania; Department of Clinical Hematology, Institute of Cancerology and Hematology, Teaching Hospital Brest, France; Department of Clinical Hematology, Institute of Cancerology and Hematology, Teaching Hospital Brest, France; Laboratory of Hematology, Teaching Hospital Brest, France; Cardiology Department, Rehabilitation Hospital, Cluj-Napoca, Romania; MD

**Keywords:** lymphomatous pericarditis, DLBCL, cardiac involvement

## Abstract

Cardiac involvement in non-Hodgkin’s lymphoma is a rare occurrence with a dismal prognosis, which may evolve with different clinical presentations, the most frequent being heart failure. Diagnosis of cardiac involvement is generally made by cardiac ultrasound. We report a case of lymphomatous pericarditis in the evolution of a non-Hodgkin’s lymphoma, diagnosed by PET-CT scan, and occurring concomitantly with complete isotopic remission of enlarged mediastinal lymph nodes following chemotherapy.

## Case report

We report on the case of a 51-year-old man diagnosed with a CD20-positive diffuse, large B-cell lymphoma (DLBCL), Ann Arbor stage IV (bone marrow), bulky (largest retroperitoneal adenopathy of 18 cm), with an international prognostic index of 4. Cytogenetic analysis showed t(14,18) and deletion of the *p53* gene. The patient was HIV negative.

At presentation, the patient had peritoneal effusion, whose cytological examination failed to prove the presence of lymphoma cells. Chemotherapy was started with one cycle of rituximab(R)-COP (cyclophosphamide, vincrsitin and prednisolone), followed by four cycles of R-VACP (rituximab plus vincristin, doxorubicin, cyclophosphamide and prednisolone), according to GOELAMS protocol, leading to complete remission, demonstrated by 18-F-FDG-PET scan.

The patient subsequently underwent autologous stem cell transplantation conditioned by BEAM 140 (BCNU, cytarabine, etoposide and melphalan); the number of re-infused CD34+ cells was 3 × 10^6^ cells/kg. Six months post autograft, he presented with weakness and malaise. The CT scan showed enlarged, compressive mediastinal lymphadenopathy. Rescue chemotherapy was initiated with two cycles of R-ESHAP (rituximab plus etoposide, cytarabine, methylprednisolone and cisplatin), with only minimal response.

**Figure 1.**
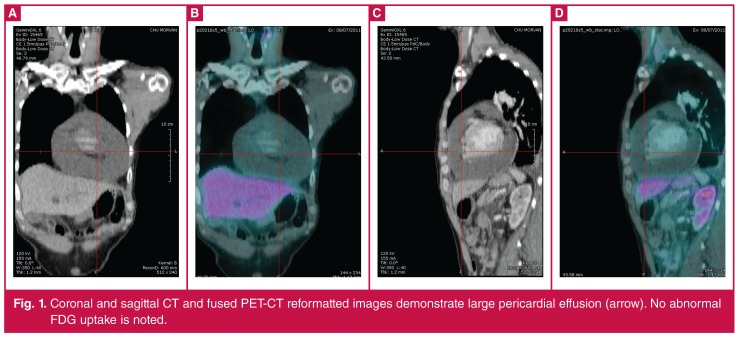
Coronal and sagittal CT and fused PET-CT reformatted images demonstrate large pericardial effusion (arrow). No abnormal FDG uptake is noted.

Because of his young age and good performance index, continuation of the rescue treatment was decided, with two courses of IVA75 (ifosfamide, etoposide and doxorubicin), leading to partial remission. Since the total anthracycline dose received to that date (490 mg/m^2^, close to the conventional maximal dose of 550 mg/m^2^) was concerning for cardiotoxicity, treatment was continued with two cycles of liposomal doxorubicin (anthracycline preparation with far lesser cardiac toxicity) and cyclophosphamide. The patient developed dyspnoea and lower leg oedema. 18-F-FDG PET scan evaluation showed complete remission of the enlarged mediastinal lymph nodes, but also a large pericardial effusion (Fig. 1).

The patient was admitted to the intensive care unit. Two-dimensional echocardiography confirmed the presence of a massive, 4-cm-thick pericardial effusion. Pericardial tap drained 1 000 ml of liquid. Cytological analysis demonstrated massive infiltrate with large CD19+ and CD5– lymphoma cells. On fluorescence in situ hybridisation analysis, these cells showed identical cytogenetic abnormalities to the original diagnosis. The presence and development of lymphomatous pericardial effusion was discordant with complete remission of the mediastinal lymph nodes observed on the PET scan.

A cycle of R-DA-EPOCH (dose-adjusted etoposide, doxorubicin, vincristine, cyclophosphamide and prednisone, plus rituximab) chemotherapy was administered. This was followed by febrile neutropenia, and death due to septic shock 13 months after the initial diagnosis.

## Discussion

Cardiac involvement in lymphomas is a rare clinical presentation with a dismal prognosis, occurring either as a primary cardiac lymphoma,^1^ or, more frequently, as secondary involvement in the late evolution of aggressive lymphomas.^2^ The clinical presentation of cardiac involvement in lymphoma is variable. Often, diagnosis is made by routine echocardiography. This is regularly performed because evaluation of cardiac function is important for assessment of the tolerability and side effects of chemotherapy, especially in anthracycline-containing regimens, which are known for their cardiotoxicity.^3^,^4^

Even if the frequency of this type of tumour appears to be increasing, real epidemiological figures are unknown, helped by the fact that involvement of the heart is often asymptomatic. Although rarely diagnosed during neoplastic clinical evolution, cardiac metastases have been found in more than 10% of post mortem examinations of patients succumbing to cancer,^5^ more frequently in melanoma, lung and breast cancer.

In lymphoma patients, post mortem figures for cardiac involvement range from nine to 20%.^5^,^6^ The diagnostic difficulty in late evolution of non-Hodgkin lymphoma is associated with the fact that heart failure may also be due to cumulative cardiac toxicity of multiple lines of treatment and of the toxic cardiac effects of anthracyclin-based chemotherapy regimens.^7^,^8^ The role of 18-F-FDG PET scans in detecting cardiac involvement of lymphoma has been described in several case reports of extralymphatic tumour involvement in lymphoma.^9^,^10^

In our patient, cardiac involvement appeared as a late evolution of an aggressive DLBCL. After four lines of chemoimmunotherapy, including autologous stem cell transplantation, the patient relapsed with mediastinal lymphadenopathy. Because of the high cumulative dose of anthracyclines, well known for their cardiotoxic effect, we chose to continue with a regimen containing less cardiotoxic pegylated anthracycline, associated with alkylating agent cyclophosphamide. After two cycles the patient developed clinical signs of right cardiac failure. Examination by PET scan found the presence of a massive pericardial effusion (Fig. 1), but discordantly, complete remission of the initial localisations of the lymphoma.

Our first hypothesis on the cause of the effusion was the cumulative toxic effect of chemotherapy, but cytology, immunophenotyping and cytogenetic analysis of the liquid obtained by puncture showed the presence of lymphoma. Lymphoproliferative disease is regarded as systemic and pericardial, i.e. extralymphatic involvement is a sign of the highest degree of dissemination in lymphoma staging, warranting systemic therapy after removal of the pericardial effusion fluid, rather than performing pericardiectomy or pericardial sclerosis.

## Conclusion

We suggest that, if signs or symptoms of cardiac failure develop during or after chemotherapy for lymphoma, the hypothesis of cardiac involvement of lymphoma should be considered. The diagnosis of this usually late complication requires cytological confirmation.
